# Access to healthcare services and adherence to treatments for people with dementia among ethnic minority groups: a scoping review

**DOI:** 10.3389/frdem.2026.1735266

**Published:** 2026-02-16

**Authors:** Elisa Aguzzoli, Magdalena Walbaum, Martin Knapp

**Affiliations:** Care Policy and Evaluation Centre, Department of Health Policy, London School of Economics and Political Science (LSE), London, United Kingdom

**Keywords:** access to care, dementia, ethnic minorities, inequalities, treatments

## Abstract

**Background:**

Dementia is a leading cause of death among the older population and requires regular engagement with primary care services, monitoring, and specialist support. People with dementia from ethnic minority groups face barriers in accessing adequate healthcare. Factors such as different conceptualisations of dementia, personal beliefs, and cultural backgrounds can influence attitudes to treatments, further increasing health inequalities and worsening health outcomes. This research maps the evidence on access and experiences within primary care services for people with dementia, along with treatment adherence patterns.

**Methods:**

A scoping review was conducted in October 2025 on Medline (Ovid), EMBASE (Ovid), and Google Scholar, with two separate searches on access to care and adherence to treatments. Eligibility criteria were peer-reviewed journal articles, published from 2010 onwards, focusing on people with dementia, and including an analysis of differences among ethnic minority groups. Studies were assessed for quality. Data were synthesised narratively.

**Results:**

Seventeen articles were included. People with dementia from ethnic minority groups report lower quality care received. They are less likely to be prescribed and receive anti-dementia medications, and they have lower adherence rates compared to their White counterparts when these medications are prescribed. Identified barriers to access and adherence to treatments include cultural factors, religious beliefs, viewing dementia as a normal part of ageing, stigma, limited language proficiency, inadequate facilities and services not tailored to the specific needs of minority ethnic groups.

**Conclusion:**

This research highlights persistent inequalities among people with dementia from ethnic minority groups. More research is needed to address these issues and fully understand the factors influencing access to care services and attitudes towards medications among people with dementia. Policymakers and researchers should emphasise the importance of designing and implementing culturally tailored interventions to reduce these persistent inequalities.

## 1 Introduction

Dementia is one of the leading causes of death in older age (WHO, 2025). People with dementia experience memory loss, changes in behaviours, reduced independence, difficulties in performing daily activities, and challenges in maintaining social relationships (Livingston et al., 2017). Due to its neurological degenerative nature, people with dementia often require periodic, if not constant, supervision, regular healthcare visits, and support from unpaid carers (Walsh et al., 2023). Managing dementia can therefore be highly challenging for both those living with the condition and their families (Browning et al., 2022). Recent research on unmet needs for people with dementia emphasises the importance of accessing comprehensive care and attending regular visits to the general practitioner (GP), as well as considering psychosocial measures aimed at reducing loneliness and social exclusion (Scharf et al., 2025). Well-structured care pathways that include regular monitoring and meaningful involvement in treatment decisions are especially valuable in the post-diagnostic period, as they can lead to better long-term outcomes (Frost et al., 2020). This requires monitoring of the progression of the condition and ensuring that treatment plans are regularly revised and tailored to the person. Despite challenges in implementation (Wheatley et al., 2021), person-centred approaches in post-diagnostic care are considered a benchmark of quality in dementia services (Bamford et al., 2021; Kim and Park, 2017).

### 1.1 Access to primary care

Primary care serves as first point of contact for people with dementia in the UK and plays a fundamental role in the management of the condition. GPs are responsible for monitoring disease progression, identifying health needs, and coordinating ongoing care. Access to primary care also enhances health literacy and the competencies of people with dementia and their carers over time (Kimzey et al., 2022). However, significant disparities persist in dementia care, particularly concerning social determinants of health, experiences with discrimination, and unequal access to GP consultations and routine primary care monitoring visits (Balls-Berry and Babulal, 2022). People with dementia from ethnic minority groups in the UK are more likely to experience barriers in accessing healthcare services. These barriers can be attributed to difficulties navigating the health system, limited health literacy, language barriers, cultural conceptualisations of dementia, stigma and perceived discrimination, denial, lack of social support and socioeconomic constraints (Hossain and Khan, 2020; Giebel et al., 2021; Sorrentino et al., 2025; Kenning et al., 2017; Stephan et al., 2018; Giebel et al., 2024).

In this review, the term ethnic minority refers to a social group with a shared cultural identity, which may include language, traditions, geographic origin, nationality, religion, customs or a combination of these (United Nations, 2018). These groups are often numerically smaller and/or socially marginalised within a larger population (Gill et al., 2018). Cultural meanings and cultural representations of dementia (Hillman and Latimer, 2017) also play significant roles in shaping perceptions of dementia. Due to its multifaceted nature and because symptoms can greatly vary between individuals, different ethnic groups may have different conceptualisations of dementia, such as viewing dementia as a normal part of ageing, often leading to delays in seeking help and getting a diagnosis, contributing to stigma, and resulting in lack of support (Hillman and Latimer, 2017; Giebel et al., 2019; ADI, 2024; Milne, 2020). Perceived discrimination, which is closely linked to medical mistrust, can further undermine service use (Lee et al., 2009; Bazargan et al., 2021), while language barriers may complicate communication and increase the risk of mistrust, miscommunication, and reduced adherence to medical advice. Such barriers perpetuate and exacerbate existing inequalities (Cooper et al., 2016).

### 1.2 Adherence to treatments

Healthcare professionals in primary care settings also play a fundamental role in dementia management, treatment monitoring, and decisions regarding medication regimes. Dementia-related symptoms, such as memory loss and decline in executive functioning can contribute to poor adherence to treatments (Kröger et al., 2017; Smith et al., 2017), which can cause negative health outcomes, including increased risk of hospitalisation or death (El-Saifi et al., 2018). Factors influencing adherence include age, type of medication, polypharmacy – defined as the regular use of several medications at the same time (Varghese et al., 2025) – and medication costs borne by the patient (El-Saifi et al., 2018). Additional barriers such as limited disease-related knowledge, low health literacy, adverse effects, weak patient-provider relationships, logistical barriers (Gellad et al., 2011), uncooperative patients (Campbell et al., 2012), comorbidities (Kremenchugsky and Wick, 2019), and lack of social support (Hudani and Rojas-Fernandez, 2016) further contribute to poor adherence.

### 1.3 Aims of the review

This review seeks to examine how access to care, particularly primary care, together with interactions with healthcare professionals, cultural and social factors, and broader inequalities in access and quality of care, influence the attitudes of people with dementia towards treatment and their behaviours around medication adherence. Despite the relevance of these factors, research directly addressing dementia medication adherence among ethnic minority groups remains limited (Arlt et al., 2008). Although access to health care is a multi-dimensional and complex concept, here it is referred to as the opportunity to obtain and utilise health care when it is wanted or needed (Gulliford et al., 2002). In line with guidance from the National Institute for Health and Care Excellence (NICE), adherence to treatments refers to the extent to which a patient’s actions matches the agreed recommendations (NICE, 2009). By synthesising the available evidence, the review aims to identify gaps in the literature and highlight potential epistemological challenges in this field.

## 2 Methods

### 2.1 Search strategy

A scoping review was conducted in October 2025 using Medline (Ovid), EMBASE (Ovid), and a targeted grey literature search through Google Scholar, restricted to English-language publications from 2010 onwards. For the targeted search through Google Scholar, we reviewed the first 10 pages, with ten records per page. To address the two main themes of the review (1. access and 2. adherence), two separate searches were performed following the PRISMA guideline. No review protocol was registered for this scoping review. Based on the Office for National Statistics (ONS) study guidelines (ONS, 2025), we avoided *race* as a search terminology since it places people into categories based on physical characteristics. Here we refer to ethnicity as a self-defined concept. For Search 1, the following terms were utilised: *Dementia, Alzheimer’s disease, Pharmacological, Medication, Therapy, Healthcare, Drug, Treatment, Access, Ethnic minority, Cultural background, Ethnicity.* For Search 2, the search strategy involved the following combination of terms: *Dementia, Treatment, Medication, Therapy, Preference, Adherence, Initiation, Uptake, Alzheimer’s disease, Ethnic minority, Cultural background, Ethnicity*. Adequate evidence was retrieved through the combination of these search terms.

### 2.2 Eligibility criteria, study selection, and data synthesis

Eligibility criteria were developed using the Population–Concept–Context (PCC) framework (JBI, 2017), with the population defined as people with dementia, the concept as differences in access to care and adherence to treatment, and the context as ethnic minority groups. Eligible studies were peer-reviewed journal articles including systematic and scoping reviews, randomised control trials (RCTs), observational studies, and qualitative studies. Studies focused on differences in dementia diagnosis rates and timing of diagnosis were excluded. Studies were selected based on relevance and appropriateness for the focus of this research. Some articles identified in Search 2 that were relevant to Search 1 were retained. Included articles were checked by a second reviewer; discrepancies were resolved through discussion. One independent reviewer extracted data from the selected studies. Study characteristics were summarised as shown in Supplementary Tables 1, 2 on access to care and adherence to treatments, respectively. Extracted data included author, year of publication, country, study design, study focus, and main findings. The data was narratively synthesised.

## 3 Results

**Figure 1:**
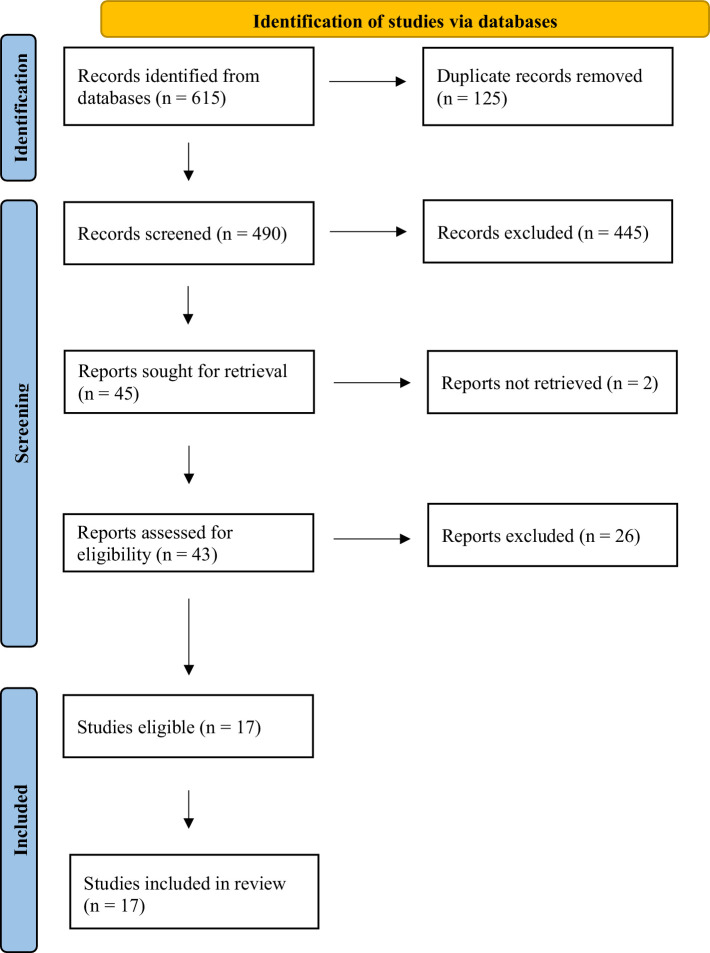
PRISMA diagram of included studies.

A total of 615 articles were retrieved (Search 1 *n* = 357 and Search 2 *n* = 258) (see Figure 1). After removing duplicates, 490 articles remained for title and abstract screening. A total of 43 articles were selected for full-text screening. After full-text review, 17 articles were retained (eleven from Search 1 and six from Search 2) (see Supplementary Tables 1, 2).

### 3.1 Access

#### 3.1.1 Dementia medication initiation

Three studies focused on anti-dementia treatment initiation (Cooper et al., 2010; Stevnsborg et al., 2016; Jones et al., 2020). One systematic review concluded that people with dementia from ethnic minority groups, once diagnosed, are less likely to access anti-dementia medications (Cooper et al., 2010). The authors argued that inequalities in accessing healthcare services and the quality of care received significantly influence access to anti-dementia treatments (Cooper et al., 2010). Similarly, a study based on a Danish registry revealed that immigrant background is associated with a significantly lower likelihood of receiving anti-dementia drug therapy (Stevnsborg et al., 2016). A UK-based study revealed that, among people with dementia, Asian people are less likely to be prescribed anti-dementia drugs when indicated and then receive them for, on average, 15 days/year less compared to people from White ethnic groups (Jones et al., 2020).

#### 3.1.2 Inequalities in access to healthcare services

Three studies examined inequalities in access to healthcare services (Albaroudi and Chen, 2022; Hinton et al., 2024; Subramaniam and Mukaetova Ladinska, 2025). A study evaluating the Consumer Assessment of Healthcare Providers and Systems (CAHPS) in the US showed significant racial and ethnic disparities in the CAHPS scores, with African American or Black, Asian, and Hispanic people with dementia reporting lower total CAHPS scores compared to White patients (Albaroudi and Chen, 2022). CAHPS measures aspects such as access to specialists, communication, educational information, timeliness, and shared decision-making. Hence, African American or Black patients who report lower CAHPS scores often experience reduced access to care, lack of trust in providers, delays in receiving care, poorer communication with providers, limited access to specialty services, and lower involvement in shared decision-making compared to White people (Albaroudi and Chen, 2022).

Similarly, a scoping review of US-based studies analysing racial and ethnic healthcare disparities for people living with dementia found that minoritised populations are less likely to be prescribed anti-dementia medications, use hospice care, and have a higher risk of hospitalisation (Hinton et al., 2024). Most studies report racial and ethnic differences not only in medication use among people with dementia, but also in the use, costs, and quality of care received (Hinton et al., 2024). A narrative review concluded that reduced access to healthcare services by people with dementia from ethnic minority groups is often a manifestation of underlying systemic disadvantages within dementia services (Subramaniam and Mukaetova Ladinska, 2025). People from ethnic minority communities experience poorer treatment outcomes due to the lack of culture-specific support and efforts by local healthcare systems to minimise some of these disadvantages (Subramaniam and Mukaetova Ladinska, 2025).

#### 3.1.3 Barriers to access healthcare services

Five studies focused on barriers to access to healthcare services (Hossain and Khan, 2020; Sorrentino et al., 2025; Nielsen et al., 2021; Mukadam et al., 2011; Mukadam et al., 2013). A qualitative study on service use conducted in Denmark highlighted the importance of examining care practises and perceived consequences of dementia in minority ethnic communities (Nielsen et al., 2021). These communities face persistent barriers to accessing dementia care services, heavily influenced by cultural factors such as limited language proficiency and strong cultural norms, including familial responsibility for the care of older family members and stigma associated with mental illness and dementia. Additionally, available services are rarely tailored to the specific needs of minority ethnic service users and are inadequate (Nielsen et al., 2021). In terms of access to dementia services for minority ethnic groups, a qualitative study conducted in England revealed that people experience barriers due to their religious and cultural beliefs as well as negative past experiences with healthcare professionals and the complexity of the healthcare system (Hossain and Khan, 2020).

A systematic review of ethnicity and pathways to care in dementia conducted by Mukadam et al. found that barriers to accessing specialist help for dementia include not conceptualising dementia as an illness, believing that dementia is a normal part of ageing, attributing dementia to spiritual, psychological, physical health or social causes, feeling that caring for the person with dementia is a personal or family responsibility, experiencing stigma within the community, and negative experiences of healthcare services (Mukadam et al., 2011). As they explained in a more recent review, Mukadam et al., showed that the three main categories of barriers to help-seeking for dementia in minority ethnic groups seem to be knowledge-related (i.e., different beliefs about the cause and knowing the purpose of a diagnosis); society-related (i.e., concern about stigma and cultural expectations of looking after your own relatives); and healthcare-related (i.e., any hesitation in approaching healthcare professionals or any barriers within the healthcare system itself) (Mukadam et al., 2013). Similarly, a systematic review about barriers to access and utilisation of dementia care services in Europe classified barriers into five categories: (1) informational and educational barriers including lack of awareness and knowledge; (2) organisational barriers involving poor care coordination and unclear access procedures; (3) cultural and stigma-related barriers linked to societal attitudes towards dementia; (4) financial barriers associated with the high costs of care; and finally (5) logistical barriers including limited availability and accessibility of support services (Sorrentino et al., 2025).

### 3.2 Adherence

#### 3.2.1 Differences among the population in adherence rates

Six studies focused on differences in adherence rates and analysed racial and ethnic differences in medication use among people with dementia (Olchanski et al., 2023; Giebel et al., 2023; Zhu et al., 2022; Thorpe et al., 2016; Pilonieta et al., 2023; Dong et al., 2024). A US-based study showed that initiation of anti-dementia medications does not vary by ethno-racial group, but non-Hispanic Black people have lower adherence compared to White people (Olchanski et al., 2023). Another US-based study found that people with dementia from minority ethnic backgrounds use memantine and Acetylcholinesterase Inhibitors (AChEIs) less frequently than those from a White ethnic background (Giebel et al., 2023). A study reported that US Black and Hispanic people remain less likely than White people to report any new AChEI or memantine treatment during follow-up. In addition, among those who reported new treatment during follow-up, Black and Hispanic participants were less likely than White participants to be persistently treated with AChEI and memantine (Zhu et al., 2022). This study showed that despite racial/ethnically under-represented populations being diagnosed at a later stage and, consequently, being more impaired at first dementia diagnosis, White people with dementia are still more likely to be treated at any severity, magnifying disparities in treatment (Zhu et al., 2022).

Three studies of differences in the population and adherence rates were conducted in the US, looking at Medicare beneficiaries with Alzheimer’s Disease and Alzheimer’s Disease-Related Dementias (ADRD). One study reported that, among US Medicare beneficiaries with ADRD, Black and Hispanic people discontinue anti-dementia drugs at a faster rate compared to White people (Thorpe et al., 2016). In accordance with these findings, another study found that Black beneficiaries had lower medication adherence compared to their White counterparts (Pilonieta et al., 2023). Lower AD medication adherence was associated with individual-level factors such as race and ethnicity, and with having more than one specialist visit (Pilonieta et al., 2023). Another study by Dong et al., taking into account multiple ethnic minority groups, revealed that Black, Hispanic, and Asian/Pacific Islander patients were more likely to be nonadherent than non-Hispanic White (White) patients in 2016 (Dong et al., 2024).

## 4 Discussion

### 4.1 Access to care services

This review highlights marked inequalities in access to healthcare services, quality of care and treatment adherence in people with dementia. These disparities are particularly evident between ethnic minority groups, who often face greater barriers in accessing services. Research shows that people with dementia among ethnic minority groups receive poorer quality healthcare, are less likely to be prescribed anti-dementia medications (Strooij et al., 2024), and have lower overall rates of anti-dementia medication use compared to White populations. Studies exploring the reasons behind the underuse of dementia services by minority ethnic groups have highlighted several barriers to help-seeking, including different understanding of dementia, cultural and social concerns, beliefs about the cause of symptoms, difficulties in navigating the health system, stigma, and previous negative experiences. Differences in how dementia is conceptualised within communities further hinder help-seeking behaviours and access to care.

These interconnected issues emphasise the need for a comprehensive framework to understand how contextual factors shape access to dementia care and exacerbate health inequalities. The Health Equity Measurement Framework (HEMF) (Dover and Belon, 2019) provides a useful conceptual basis for understanding how factors such as cultural background, beliefs, trust, and structural barriers to health equity influence access to primary care and medication adherence. The framework not only situates these factors within broader contexts but also considers other important factors that impact equity in access to care (and treatment adherence) such as health behaviours, health beliefs, environment, perceived need, utilisation of health-promoting resources, appropriateness, and healthcare utilisation (Dover and Belon, 2019). Further research is needed to elucidate the role that ethnicity, culture, and these additional factors play in the help-seeking pathway for dementia and to design interventions to improve equity of access to healthcare services.

### 4.2 Adherence to treatments

As several studies show, ethnic disparities in the uptake and persistence of treatment remain even in controlled settings of longitudinal research studies. These differences often have a crucial impact on health outcomes and result in a greater burden of dementia, particularly if untreated or treated only in the later stages. Individuals’ characteristics and beliefs about medicines influence decisions over the uptake of the medications (Munoz et al., 2023). Often people with dementia prioritise their quality of life, self-efficacy (Lepper et al., 2020), and prefer retaining autonomy even when this means accepting the risks associated with not adhering to prescribed medications (Rees et al., 2020). Moreover, factors such as patient refusal, early disease stage (Podhorna et al., 2020), age, sex, socioeconomic status, costs, as well as the extent and quality of interactions among patients, caregivers, and providers, also influence persistence with anti-dementia treatments (Maxwell et al., 2014). Among ethnic minorities, the use of traditional remedies (WHO, 2023) (for example, medicinal plants traditionally used to treat dementia) (Tewari et al., 2018) may reflect distrust of Western medicine and may contribute to the avoidance of prescription medications recommended by general practitioners (Agu et al., 2019).

Barriers to treatment adherence include passive resistance, which may manifest as refusal to initiate treatment (Dooley et al., 2019), alongside limited knowledge, stigma, beliefs about medication efficacy, illness perceptions (such as perceiving dementia as a normal part of ageing), and trust in GPs (Chia et al., 2006). These factors not only significantly influence people’s attitudes and behaviours but also constitute significant barriers to anti-dementia medication adherence and health-seeking behaviours, highlighting the need for further investigation. Evidence highlights persistent inequalities in adherence to dementia treatments and suggests that issues related to reduced treatment uptake among people with dementia is worth exploring. A deeper exploration of the effect of belief-laden variables including self-efficacy and illness perceptions on adherence patterns (Chia et al., 2006) could also help assess whether the benefits of dementia medications have been underestimated due to inconsistent use. Understanding and addressing these overlooked disparities is crucial for informing policy and designing targeted interventions aimed at reducing inequalities and improving treatment adherence across all groups.

### 4.3 Review limitations

This review focused on studies involving people with a formal diagnosis of dementia. Consequently, factors influencing disparities around diagnostic timing and health-seeking behaviours (e.g., accessing diagnostic services and obtaining a diagnosis) are not addressed, as they fall outside the scope of this investigation. The decision to prioritise studies involving people with a formal dementia diagnosis is based on the assumption that disparities in diagnostic timing have a weaker correlation with health-seeking behaviours and attitudes towards treatments. Nevertheless, people living with dementia who have not yet received a formal diagnosis face challenges and disparities that need further exploration through future research.

### 4.4 Gaps in the literature

Several literature gaps have been identified. First, there is limited research on how broader social environments – including cultural background, family dynamics, personal values, comorbidities, and lived experiences in care – interact to influence access. Existing studies tend to focus narrowly on service availability or quality of care, neglecting this wider context. To better understand barriers to accessing care, a more comprehensive approach is needed. Recent research has increasingly advocated for the analysis of group-specific cultural values, the provision of adequate support for ethnic minority groups, and the development of culturally appropriate interventions. Recent studies have also highlighted a number of culturally tailored interventions targeting carers of people with dementia, while very little evidence refers to interventions for people with dementia (James et al., 2020; Lim, 2024; Assfaw et al., 2025). One study evaluated a culturally tailored dementia education programme delivered to minority ethnic communities in Denmark, aiming to improve knowledge about dementia and engagement with services (Nielsen et al., 2022). These interventions demonstrated short-term improvements in dementia awareness and help-seeking intentions, although evidence on longer-term impacts on service use or treatment adherence was limited (Nielsen et al., 2022).

Second, evidence on treatment adherence is limited. Most research concentrates on non-adherence linked to cognitive decline and the development of tools to mitigate these challenges. There is little exploration of how attitudes, cultural values, and ethnicity shape adherence behaviours in diverse populations. This gap is particularly concerning given the role of beliefs, trust, and stigma in shaping medication use. Addressing these issues is vital for the development of culturally appropriate, equitable dementia care strategies.

## 5 Conclusion

This scoping review highlights that people with dementia from ethnic minority groups face persistent and systemic inequalities in accessing adequate care services and are generally less likely to adhere to dementia treatments. These disparities are shaped by a complex interplay of factors operating across multiple contextual levels, including societal, cultural, economic, political, and individual domains. Barriers to access and treatment uptake are not only heterogeneous and multifaceted but also deeply embedded within broader structural inequities. More research is needed to address these issues and better understand the factors influencing access to care services and attitudes towards medications among people with dementia. Policymakers and researchers should emphasise the importance of designing and implementing culturally tailored interventions to reduce these persistent inequalities.

## References

[ref1] ADI (2024). World Alzheimer report 2024: Global changes in attitudes to dementia. Alzheimer’s Disease International (ADI): London, UK.

[ref2] Agu J. C. Hee-Jeon Y. Steel A. Adams J. (2019). A systematic review of traditional, complementary and alternative medicine use amongst ethnic minority populations: a focus upon prevalence, drivers, integrative use, health outcomes, referrals and use of information sources. J. Immigr. Minor. Health 21, 1137–1156. doi: 10.1007/s10903-018-0832-4, 30382488

[ref3] Albaroudi A. Chen J. (2022). Consumer assessment of healthcare providers and systems among racial and ethnic minority patients with Alzheimer disease and related dementias. JAMA Netw. Open 5:e2233436. doi: 10.1001/jamanetworkopen.2022.33436 36166229 PMC9516284

[ref4] Arlt S. Lindner R. Rösler A. von Renteln-Kruse W. (2008). Adherence to medication in patients with dementia. Drugs Aging 25, 1033–1047. doi: 10.2165/0002512-200825120-00005, 19021302

[ref5] Assfaw A. D. Reinschmidt K. M. Teasdale T. A. Stephens L. Kleszynski K. L. Dwyer K. (2025). Assessing culturally tailored dementia interventions to support informal caregivers of people living with dementia (PLWD): a scoping review. J. Racial Ethn. Health Disparities 12, 1526–1543. doi: 10.1007/s40615-024-01985-3, 38546946

[ref6] Balls-Berry J. J. E. Babulal G. M. (2022). Health disparities in dementia. Continuum (Minneap Minn) 28, 872–884. doi: 10.1212/CON.0000000000001088, 35678407 PMC9924306

[ref7] Bamford C. Wheatley A. Brunskill G. Booi L. Allan L. Banerjee S. . (2021). Key components of post-diagnostic support for people with dementia and their carers: a qualitative study. PLoS One 16:e0260506. doi: 10.1371/journal.pone.0260506, 34928972 PMC8687564

[ref8] Bazargan M. Cobb S. Assari S. (2021). Discrimination and medical mistrust in a racially and ethnically diverse sample of California adults. Ann. Fam. Med. 19, 4–15. doi: 10.1370/afm.2632, 33431385 PMC7800756

[ref9] Browning J. A. Tsang C. C. S. Dong X. Wan J. Y. Chisholm-Burns M. A. Finch C. K. . (2022). Effects of Medicare comprehensive medication review on racial/ethnic disparities in nonadherence to statin medications among patients with Alzheimer’s disease: an observational analysis. BMC Health Serv. Res. 22:159. doi: 10.1186/s12913-022-07483-8, 35130899 PMC8822650

[ref10] Campbell N. L. Boustani M. A. Skopelja E. N. Gao S. Unverzagt F. W. Murray M. D. (2012). Medication adherence in older adults with cognitive impairment: a systematic evidence-based review. Am. J. Geriatr. Pharmacother. 10, 165–177. doi: 10.1016/j.amjopharm.2012.04.004, 22657941

[ref11] Chia L. Schlenk E. A. Dunbar-Jacob J. (2006). Effect of personal and cultural beliefs on medication adherence in the elderly. Drugs Aging 23, 191–202. doi: 10.2165/00002512-200623030-00002, 16608375

[ref12] Cooper C. Lodwick R. Walters K. Raine R. Manthorpe J. Iliffe S. . (2016). Inequalities in receipt of mental and physical healthcare in people with dementia in the UK. Age Ageing 46, 393–400. doi: 10.1093/ageing/afw208 PMC5378291 27916749

[ref13] Cooper C. Tandy A. R. Balamurali T. B. S. Livingston G. (2010). A systematic review and Meta-analysis of ethnic differences in use of dementia treatment, care, and research. Am. J. Geriatr. Psychiatry 18, 193–203. doi: 10.1097/JGP.0b013e3181bf9caf, 20224516

[ref14] Dong X. Tsang C. C. S. Wan J. Y. Chisholm-Burns M. A. Finch C. K. Tsao J. W. . (2024). Effects of Medicare part D medication therapy management on racial/ethnic disparities in adherence to antidementia medications among patients with Alzheimer's disease and related dementias: an observational study. Explor. Res. Clin. Soc. Pharm. 13:100420. doi: 10.1016/j.rcsop.2024.100420 38420610 PMC10900920

[ref15] Dooley J. Bass N. Livingston G. McCabe R. (2019). Involving patients with dementia in decisions to initiate treatment: effect on patient acceptance, satisfaction and medication prescription. Br. J. Psychiatry 214, 213–217. doi: 10.1192/bjp.2018.201, 30269695

[ref16] Dover D. C. Belon A. P. (2019). The health equity measurement framework: a comprehensive model to measure social inequities in health. Int. J. Equity Health 18:36. doi: 10.1186/s12939-019-0935-0, 30782161 PMC6379929

[ref17] El-Saifi N. Moyle W. Jones C. Tuffaha H. (2018). Medication adherence in older patients with dementia: a systematic literature review. J. Pharm. Pract. 31, 322–334. doi: 10.1177/0897190017710524, 28539102

[ref18] Frost R. Walters K. Aw S. Brunskill G. Wilcock J. Robinson L. . (2020). Effectiveness of different post-diagnostic dementia care models delivered by primary care: a systematic review. Br. J. Gen. Pract. 70, e434–e441. doi: 10.3399/bjgp20x710165, 32424049 PMC7239042

[ref19] Gellad W. F. Grenard J. L. Marcum Z. A. (2011). A systematic review of barriers to medication adherence in the elderly: looking beyond cost and regimen complexity. Am. J. Geriatr. Pharmacother. 9, 11–23. doi: 10.1016/j.amjopharm.2011.02.004 21459305 PMC3084587

[ref20] Giebel C. Cations M. Draper B. Komuravelli A. (2023). Ethnic disparities in the uptake of anti-dementia medication in young and late-onset dementia. Int. Psychogeriatr. 35, 381–390. doi: 10.1017/s1041610220000794, 32484120

[ref21] Giebel C. Hanna K. Watson J. Faulkner T. O’Connell L. Smith S. . (2024). A systematic review on inequalities in accessing and using community-based social care in dementia. Int. Psychogeriatr. 36, 540–563. doi: 10.1017/S104161022300042X 37170588

[ref22] Giebel C. Robertson S. Beaulen A. Zwakhalen S. Allen D. Verbeek H. (2021). “Nobody seems to know where to even turn to”: barriers in accessing and utilising dementia care services in England and the Netherlands. Int. J. Environ. Res. Public Health 18:12233. doi: 10.3390/ijerph182212233, 34831989 PMC8622725

[ref23] Giebel C. M. Warden A. Challis D. Jolley D. Bhui K. S. Lambat A. . (2019). Age, memory loss and perceptions of dementia in south Asian ethnic minorities. Aging Ment. Health 23, 173–182. doi: 10.1080/13607863.2017.1408772 29206481

[ref24] Gill P. S. Kai J. Bhopal R. S. Wild S. (2018). “Black and minority ethnic groups” in Health care needs assessment (London: CRC Press), 227–400.

[ref25] Gulliford M. Figueroa-Munoz J. Morgan M. Hughes D. Gibson B. Beech R. . (2002). What does 'access to health care' mean? J. Health Serv. Res. Policy 7, 186–188. doi: 10.1258/135581902760082517, 12171751

[ref26] Hillman A. Latimer J. (2017). Cultural representations of dementia. PLoS Med. 14:e1002274. doi: 10.1371/journal.pmed.1002274, 28350889 PMC5370114

[ref27] Hinton L. Tran D. Peak K. Meyer O. L. Quiñones A. R. (2024). Mapping racial and ethnic healthcare disparities for persons living with dementia: a scoping review. Alzheimers Dement. 20, 3000–3020. doi: 10.1002/alz.13612, 38265164 PMC11032576

[ref28] Hossain M. Z. Khan H. T. A. (2020). Barriers to access and ways to improve dementia services for a minority ethnic group in England. J. Eval. Clin. Pract. 26, 1629–1637. doi: 10.1111/jep.13361, 32022982

[ref29] Hudani Z. K. Rojas-Fernandez C. H. (2016). A scoping review on medication adherence in older patients with cognitive impairment or dementia. Res. Social Adm. Pharm. 12, 815–829. doi: 10.1016/j.sapharm.2015.11.011, 26797263

[ref30] James T. Ceballos S. G. Mukadam N. Livingston G. (2020). A systematic review of culturally tailored dementia interventions for minority ethnic groups and low- and middle-income country populations: acceptability, feasibility and outcomes. Alzheimers Dement. 16:e039095. doi: 10.1002/alz.039095

[ref31] JBI (2017) in Reviewer's Manual. ed. Aromataris E. M. Z. (Adelaide: Joanna Briggs Institute).

[ref32] Jones M. E. Petersen I. Walters K. Bhanu C. Manthorpe J. Raine R. . (2020). Differences in psychotropic drug prescribing between ethnic groups of people with dementia in the United Kingdom. Clin. Epidemiol. 12, 61–71. doi: 10.2147/CLEP.S222126, 32021472 PMC6980848

[ref33] Kenning C. Daker-White G. Blakemore A. Panagioti M. Waheed W. (2017). Barriers and facilitators in accessing dementia care by ethnic minority groups: a meta-synthesis of qualitative studies. BMC Psychiatry 17:316. doi: 10.1186/s12888-017-1474-0, 28854922 PMC5577676

[ref34] Kim S. K. Park M. (2017). Effectiveness of person-centered care on people with dementia: a systematic review and meta-analysis. Clin. Interv. Aging 12, 381–397. doi: 10.2147/CIA.S117637, 28255234 PMC5322939

[ref35] Kimzey M. Howe C. J. Martin C. McLarty J. Baucham R. (2022). Development of health literacy in persons and caregivers living with dementia: a qualitative directed content analysis. Dementia 21, 540–555. doi: 10.1177/14713012211049691, 34654330

[ref36] Kremenchugsky S. Wick J. Y. (2019). Medication safety, adherence, and deprescribing in patients with dementia. Sr. Care Pharm. 34, 351–362. doi: 10.4140/TCP.n.2019.351, 31164182

[ref37] Kröger E. Tatar O. Vedel I. Giguère A. M. C. Voyer P. Guillaumie L. . (2017). Improving medication adherence among community-dwelling seniors with cognitive impairment: a systematic review of interventions. Int. J. Clin. Pharm. 39, 641–656. doi: 10.1007/s11096-017-0487-6, 28555421

[ref38] Lee C. Ayers S. L. Kronenfeld J. J. (2009). The association between perceived provider discrimination, healthcare utilization and health status in racial and ethnic minorities. Ethn. Dis. 19, 330–337. Available online at: https://pmc.ncbi.nlm.nih.gov/articles/PMC2750098/, 19769017 PMC2750098

[ref39] Lepper S. Rädke A. Wehrmann H. Michalowsky B. Hoffmann W. (2020). Preferences of cognitively impaired patients and patients living with dementia: a systematic review of quantitative patient preference studies. J Alzheimer's Dis 77, 885–901. doi: 10.3233/JAD-191299 32741807

[ref40] Lim J. N. (2024). “Developing culturally appropriate dementia interventions for people from culturally diverse backgrounds” in Design for dementia, mental health and wellbeing. eds. Niedderer, K., Ludden, G., Dening, T., Holthoff-Detto, V., (London: Routledge), 285–303.

[ref41] Livingston G. Sommerlad A. Orgeta V. Costafreda S. G. Huntley J. Ames D. . (2017). Dementia prevention, intervention, and care. Lancet 390, 2673–2734. doi: 10.1016/S0140-6736(17)31363-6 28735855

[ref42] Maxwell C. J. Stock K. Seitz D. Herrmann N. (2014). Persistence and adherence with dementia pharmacotherapy: relevance of patient, provider, and system factors. Can. J. Psychiatr. 59, 624–631. doi: 10.1177/070674371405901203, 25702361 PMC4304581

[ref43] Milne A. (2020). “Conceptualising dementia” in Mental health in later life: Taking a life course approach. ed. Milne A. (Bristol: Bristol University Press), 165–198.

[ref44] Mukadam N. Cooper C. Livingston G. (2011). A systematic review of ethnicity and pathways to care in dementia. Int. J. Geriatr. Psychiatry 26, 12–20. doi: 10.1002/gps.2484, 21157846

[ref45] Mukadam N. Cooper C. Livingston G. (2013). Improving access to dementia services for people from minority ethnic groups. Curr. Opin. Psychiatry 26, 409–414. doi: 10.1097/YCO.0b013e32835ee668, 23454888 PMC4222802

[ref46] Munoz S. R. de Vries S. T. Lankester G. Pignatti F. van Munster B. C. Radford I. . (2023). Preferences about future Alzheimer's disease treatments elicited through an online survey using the threshold technique. J. Prev Alzheimers Dis. 10, 756–764. doi: 10.14283/jpad.2023.84, 37874097

[ref47] NICE (2009). Medicines Adherence: Involving Patients in Decisions About Prescribed Medicines and Supporting Adherence: National Institute for health and care excellence. Available online at: https://www.nice.org.uk/guidance/cg76/chapter/Introduction (Accessed October 15, 2025). 39480983

[ref48] Nielsen T. R. Nielsen D. S. Waldemar G. (2021). Barriers in access to dementia care in minority ethnic groups in Denmark: a qualitative study. Aging Ment. Health 25, 1424–1432. doi: 10.1080/13607863.2020.1787336 32619352

[ref49] Nielsen T. R. Nielsen D. S. Waldemar G. (2022). Feasibility of a culturally tailored dementia information program for minority ethnic communities in Denmark. Int. J. Geriatr. Psychiatry 37, 1–8. doi: 10.1002/gps.5656 PMC9298896 34762345

[ref50] Olchanski N. Daly A. T. Zhu Y. Breslau R. Cohen J. T. Neumann P. J. . (2023). Alzheimer's disease medication use and adherence patterns by race and ethnicity. Alzheimers Dement. 19, 1184–1193. doi: 10.1002/alz.12753, 35939325 PMC9905357

[ref51] ONS. (2025). Ethnicity and race: Office for National Statistics; [service manual]. Available online at: https://service-manual.ons.gov.uk/content/language/ethnicity-and-race (Accessed January 6, 2026).

[ref52] Pilonieta G. Pisu M. Martin R. C. Shan L. Kennedy R. E. Oates G. . (2023). Specialist availability and drug adherence in older adults with dementia across regions of the United States. J Alzheimer's Dis 93, 927–937. doi: 10.3233/JAD-220620, 37125546 PMC10634245

[ref53] Podhorna J. Winter N. Zoebelein H. Perkins T. (2020). Alzheimer’s treatment: real-world physician behavior across countries. Adv. Ther. 37, 894–905. doi: 10.1007/s12325-019-01213-z, 31933052 PMC7004436

[ref54] Rees J. L. Burton A. Walters K. R. Leverton M. Rapaport P. Herat Gunaratne R. . (2020). Exploring how people with dementia can be best supported to manage long-term conditions: a qualitative study of stakeholder perspectives. BMJ Open 10:e041873. doi: 10.1136/bmjopen-2020-041873, 33033103 PMC7545621

[ref55] Scharf A. Michalowsky B. Rädke A. Kleinke F. Schade S. Platen M. . (2025). Identifying and addressing unmet needs in dementia: the role of care access and psychosocial support. Int. J. Geriatr. Psychiatry 40:e70066. doi: 10.1002/gps.70066, 40148225 PMC11949772

[ref56] Smith D. Lovell J. Weller C. Kennedy B. Winbolt M. Young C. . (2017). A systematic review of medication non-adherence in persons with dementia or cognitive impairment. PLoS One 12:e0170651. doi: 10.1371/journal.pone.0170651, 28166234 PMC5293218

[ref57] Sorrentino M. Fiorilla C. Mercogliano M. Stilo I. Esposito F. Moccia M. . (2025). Barriers for access and utilization of dementia care services in Europe: a systematic review. BMC Geriatr. 25:162. doi: 10.1186/s12877-025-05805-z, 40065204 PMC11892202

[ref58] Stephan A. Bieber A. Hopper L. Joyce R. Irving K. Zanetti O. . (2018). Barriers and facilitators to the access to and use of formal dementia care: findings of a focus group study with people with dementia, informal carers and health and social care professionals in eight European countries. BMC Geriatr. 18:131. doi: 10.1186/s12877-018-0816-1, 29866102 PMC5987478

[ref59] Stevnsborg L. Jensen-Dahm C. Nielsen T. R. Gasse C. Waldemar G. (2016). Inequalities in access to treatment and Care for Patients with dementia and immigrant background: a Danish Nationwide study. J Alzheimer's Dis 54, 505–514. doi: 10.3233/JAD-160124, 27567820

[ref60] Strooij B. T. Blom M. T. van Hout H. P. J. Maarsingh O. R. Elders P. J. M. van Campen J. . (2024). Potentially inappropriate medication in older persons with dementia: does a migration background matter? J. Am. Med. Dir. Assoc. 25:105150. doi: 10.1016/j.jamda.2024.105150, 39009066

[ref61] Subramaniam H. Mukaetova Ladinska E. B. (2025). Systemic disadvantages facing UK ethnic elders within dementia healthcare. BJPsych Adv. 31, 311–320. doi: 10.1192/bja.2025.10115

[ref62] Tewari D. Stankiewicz A. M. Mocan A. Sah A. N. Tzvetkov N. T. Huminiecki L. . (2018). Ethnopharmacological approaches for dementia therapy and significance of natural products and herbal drugs. Front. Aging Neurosci. 10:3. doi: 10.3389/fnagi.2018.00003 29483867 PMC5816049

[ref63] Thorpe C. T. Fowler N. R. Harrigan K. Zhao X. Kang Y. Hanlon J. T. . (2016). Racial and ethnic differences in initiation and discontinuation of antidementia drugs by Medicare beneficiaries. J. Am. Geriatr. Soc. 64, 1806–1814. doi: 10.1111/jgs.14403, 27549029 PMC5026892

[ref64] United Nations (2018). The report on the world social situation 2018. New York: United Nations.

[ref65] Varghese D. Ishida C. Patel P. Haseer Koya H. (2025). “Polypharmacy” in StatPearls. ed. Shams P. (Treasure Island (FL): StatPearls PublishingCopyright © 2025, StatPearls Publishing LLC).

[ref66] Walsh S. Govia I. Peters R. Richard E. Stephan B. C. M. Wilson N.-A. . (2023). What would a population-level approach to dementia risk reduction look like, and how would it work? Alzheimers Dement. 19, 3203–3209. doi: 10.1002/alz.12985 36791256

[ref67] Wheatley A. Bamford C. Brunskill G. Booi L. Dening K. H. Robinson L. (2021). Implementing post-diagnostic support for people living with dementia in England: a qualitative study of barriers and strategies used to address these in practice. Age Ageing 50, 2230–2237. doi: 10.1093/ageing/afab114, 34240114 PMC8675435

[ref68] WHO (2023). Traditional Medicine has a Long History of Contributing to Conventional Medicine and Continues to Hold Promise. World Health Organization. Available online at: https://www.who.int/news-room/feature-stories/detail/traditional-medicine-has-a-long-history-of-contributing-to-conventional-medicine-and-continues-to-hold-promise (Accessed October 15, 2025).

[ref69] WHO. (2025) Dementia. Available online at: https://www.who.int/news-room/fact-sheets/detail/dementia (Accessed January 6, 2026).

[ref70] Zhu C. W. Neugroschl J. Barnes L. L. Sano M. (2022). Racial/ethnic disparities in initiation and persistent use of anti-dementia medications. Alzheimers Dement. 18, 2582–2592. doi: 10.1002/alz.12623, 35218291 PMC9402814

