# Correction: Access to healthcare services and adherence to treatments for people with dementia among ethnic minority groups: a scoping review

**DOI:** 10.3389/frdem.2026.1886386

**Published:** 2026-06-10

**Authors:** Elisa Aguzzoli, Magdalena Walbaum, Martin Knapp

**Affiliations:** Care Policy and Evaluation Centre, Department of Health Policy, London School of Economics and Political Science (LSE), London, United Kingdom

**Keywords:** access to care, dementia, ethnic minorities, inequalities, treatments

The reference for NICE. (2009) was erroneously written as NICE. (2009) Medicines adherence: involving patients in decisions about prescribed medicines and supporting adherence: National Institute for health and care excellence. Available online at: https://www.nice.org.uk/guidance/cg76/chapter/Introduction. (Accessed October 15, 2026).

It should be NICE (2009). *Medicines Adherence: Involving Patients in Decisions About Prescribed Medicines and Supporting Adherence: National Institute for health and care excellence*. Available online at: https://www.nice.org.uk/guidance/cg76/chapter/Introduction (Accessed October 15, 2025).

The reference for WHO. (2023) was erroneously written as WHO. (2023) Traditional medicine has a long history of contributing to conventional medicine and continues to hold promise. World Health Organization. Available online at: https://www.who.int/news-room/feature-stories/detail/traditional-medicine-has-a-long-history-of-contributing-to-conventional-medicine-and-continues-to-hold-promise (Accessed October 15, 2026).

It should be WHO (2023). *Traditional Medicine has a Long History of Contributing to Conventional Medicine and Continues to Hold Promise*. World Health Organization. Available online at: https://www.who.int/news-room/feature-stories/detail/traditional-medicine-has-a-long-history-of-contributing-to-conventional-medicine-and-continues-to-hold-promise (Accessed October 15, 2025).

The original version of this article has been updated.

