# Supplementary material for: Access to healthcare services and adherence to treatments for people with dementia among ethnic minority groups: a scoping review
Source: Front Dement. 2026 Feb 16;5:1735266. doi: 10.3389/frdem.2026.1735266 (PMC12950575; doi:10.3389/frdem.2026.1735266)
Supplement: Supplementary file 1 [file Table_1.docx]

Supplementary Table 1. Characteristics of studies included in the scoping review on access to care for people with dementia (Search 1).

| **Author** | **Year** | **Country** | **Study design** | **Study focus** | **Main findings** |
| --- | --- | --- | --- | --- | --- |
| Cooper et al | 2010 | US, Australia, UK | Systematic review and meta-analysis | Access to dementia services for people with dementia from ethnic minority (EM) groups | African Americans are 30% less likely to be prescribed cholinesterase inhibitors (odds ratio (OR) 0.7, 95% CI 0.6–0.9; z=-3.1, p=0.002; N=175) |
| Stevnsborg et al | 2016 | Denmark | Cross-sectional register-based study | Inequalities of access to anti-dementia treatment and care between immigrant and Danish-born patients with dementia | Immigrant background was associated with a significantly lower likelihood of receiving anti-dementia drug therapy (OR non-Western = 0.70, 95% CI 0.56–0.87; Western = 0.74, 95% CI 0.63–0.87) |
| Jones et al | 2020 | UK | Retrospective cohort study using UK primary care electronic health records | Psychotropic drug prescribing initiation and duration between people with dementia from White, Black, and Asian ethnic groups | Compared to White ethnic groups, Asian people were less likely to be prescribed anti-dementia drugs when indicated (adjusted prevalence rate ratio 0.86, 95% CI 0.76–0.98) and received them for on average 15 days/year less. Among individuals who were prescribed antipsychotic drugs, Asian and Black individuals had them prescribed for 17 and 27 days/year more, respectively (190.8, 95% CI 179.6–199.1 and 200.7 95% CI 191.1–206.5 days) compared to White groups |
| Albaroudi et al | 2022 | US | Cross-sectional analysis | Racial and ethnic disparities in Consumer Assessment of Healthcare Providers and Systems (CAHPS) among patients with Alzheimer disease and related dementias (ADRD) | Compared with their White counterparts, African American or Black (−1.05; 95% CI −1.15−0.95; P < .001), Asian (−0.414; 95% CI −0.623−0.205; P < .001), and Hispanic (−0.099; 95% CI −0.229 to 0.032; P = .14) patients with ADRD reported lower total CAHPS scores |
| Hinton et al | 2024 | N/A | Scoping review | Disparities in AD healthcare, including access, quality, and outcomes for ethnic minority persons living with dementia | Minoritised populations are less likely to receive an accurate and timely diagnosis, be prescribed anti‐dementia medications, and receive more aggressive life‐sustaining treatment at the end of life |
| Subramaniam et al | 2025 | UK | Narrative review | Health inequalities in dementia care experienced by ethnic minority groups | People from ethnic minority groups in the UK face systemic disadvantages in dementia healthcare, including lower access to memory services and poorer treatment outcomes compared with White British counterparts. Ethnic minority groups encounter cultural, linguistic, and structural barriers which contribute to under-utilisation of services and inequities in care |
| Hossain et al | 2020 | UK | Qualitative study | Barriers to health care service use among people with dementia from the Bangladeshi community in the UK | People from the Bangladeshi community experience multiple barriers to accessing dementia services, including religious and cultural beliefs, complexities in the healthcare system, and gender-related caregiving norms |
| Sorrentino et al | 2025 | Europe | Systematic review | Barriers to access and utilisation of dementia care services | Barriers to dementia care are categorised into information, organisational, cultural and stigma-related, financial, and logistical challenges |
| Nielsen et al | 2021 | Denmark | Qualitative study | Barriers in access to dementia care in Turkish, Pakistani and Arabic speaking minority ethnic groups | Barriers in access to dementia care are related to lacking language proficiency and strong cultural norms, including familial responsibility for the care of older family members and stigma associated with mental illness and dementia |
| Mukadam et al | 2011 | N/A | Systematic review | Pathways to dementia specialist care in EM groups | Barriers to accessing specialist help for dementia included: not conceptualising dementia as an illness; believing dementia was a normal consequence of ageing; thinking dementia had spiritual, psychological, physical health or social causes; experiences of shame and stigma within the community; and negative experiences of healthcare services |
| Mukadam et al | 2013 | UK | Narrative review | Underuse of dementia services by minority ethnic groups and barriers to help-seeking | People from minority ethnic groups are less likely to access services for dementia and do so at a later stage. This is due to cultural beliefs, stigma, low awareness, and healthcare system barriers |

US=United states, UK=United Kingdom, EM=Ethnic minority, OR=Odds ratio, CI=Confidence interval, CAHPS=Consumer Assessment of Healthcare Providers and Systems, AD=Alzheimer's disease, ARDR= Alzheimer disease and related dementias
