# Supplementary material for: Access to healthcare services and adherence to treatments for people with dementia among ethnic minority groups: a scoping review
Source: Front Dement. 2026 Feb 16;5:1735266. doi: 10.3389/frdem.2026.1735266 (PMC12950575; doi:10.3389/frdem.2026.1735266)
Supplement: Supplementary file 2 [file Table_2.docx]

Supplementary Table 2. Characteristics of studies included in the scoping review on adherence to treatments for people with dementia (Search 2).

| **Author** | **Year** | **Country** | **Study design** | **Study focus** | **Main findings** |
| --- | --- | --- | --- | --- | --- |
| Olchanski et al | 2023 | US | Retrospective cohort study | Racial and ethnic differences in medication utilisation for US people with ADRD | Non-Hispanic Black people were more likely than White people to not adhere to ADRD medication therapy (OR 1.50, 95% CI 1.07–2.09) |
| Giebel et al | 2023 | US | Cross-sectional longitudinal cohort study | Ethnicity and anti-dementia medication uptake | People with dementia from minority ethnic backgrounds used memantine and cholinesterase inhibitors less often than those from a White ethnic background |
| Zhu et al | 2022 | US | Longitudinal cohort study | Acetylcholinesterase (AChEI) treatment prescriptions during follow-up, and persistence of treatment during follow-up | Black/African American and Hispanic participants remained less likely than White participants to report any new AChEI or memantine treatment during follow-up. Among those who reported new treatment during follow-up, both Black/African American and Hispanic participants were less likely than White participants to be persistently treated with AChEI and memantine |
| Thorp et al | 2016 | US | Retrospective cohort study | Medication initiation and time to discontinuation of antidementia drugs across ethnic groups | Hispanic Medicare beneficiaries were more likely than White beneficiaries to initiate (adjusted OR 1.25, 95% CI 1.10-1.41). Hispanic and Black beneficiaries discontinued at a faster rate than White beneficiaries (adjusted hazard ratio [HR] 1.56, 95% CI 1.34-1.82 and HR=1.25, 95% CI 1.08-1.44, respectively) |
| Pilonieta et al | 2023 | US | Retrospective cohort study | Medication adherence among Medicare beneficiaries with dementia or ADRD | Race and having ≥1 specialist visits were associated with a lower risk of adherence in both regions (p <.0001) |
| Dong et al | 2024 | US | Retrospective cohort study | Ethnic disparities in the adherence to antidementia medications among patients with ADRD | Black, Hispanic, and Asian/Pacific Islander patients were more likely to be nonadherent than non-Hispanic White patients |

US=United states, OR=Odds ratio, CI=Confidence interval, ARDR= Alzheimer disease and related dementias, AChEI=Acetylcholinesterase
